# Associations of Sensory and Motor Functions With Neuropathological Changes in Aging

**DOI:** 10.1093/geroni/igaf122.1078

**Published:** 2025-12-31

**Authors:** Jeannette Mahoney, Jennifer Deal, Natascha Merten

## Abstract

Alzheimer’s disease (AD) is the most-common cause of dementia, affecting nearly 6.7 million older adults in America. The number of people with AD is projected to increase two-fold by 2050, potentially reaching 13.8 million. While AD is characterized by cognitive impairments as well as mobility disorders, it also alters sensory processing as it associated with accumulation of proteins (plaques) and tangles, which disrupt communication between brain cells. Such disruption in neural signaling leads to sensory integration issues that often precede cognitive decline. In fact, growing evidence suggests that Alzheimer’s pathology manifests in sensory association areas well before appearing in neural regions involved in higher‐order cognitive areas like the prefrontal cortex. As well, impairments in olfaction (loss of smell) are considered an early warning sign of Alzheimer’s onset. While evidence suggests that functional changes in both sensory and motor systems modulate the progression of Alzheimer’s disease, research is still in its infancy. In what follows, we will present findings from four distinct research studies examining links between sensory (auditory and olfaction) and motor (gait speed & endurance) impairments and cognitive impairments and AD biomarkers. Identification of novel, non-cognitive, non-invasive biomarkers of Alzheimer’s disease and Alzheimer’s-related dementias are a national priority identified by the NIH. Our continued research efforts are directly in line with this goal of identifying AD earlier and intervening as soon as possible in an effort to reduce the burden of Alzheimer’s disease on individuals, their families, and our society at large. Sensory Health Interest Group Sponsored Symposium

